# Isolation of Anaerobic Bromate-Reducing Bacteria Using Different Carbon Sources and Transcriptomic Insights From *Klebsiella variicola* Glu3

**DOI:** 10.3389/fmicb.2022.851844

**Published:** 2022-03-29

**Authors:** Dan Wang, Yicheng Wang, Xinyue Lv, Xunchao Cai, Waheed Iqbal, Bo Yang, Dan Zhou, Christopher Rensing, Yanping Mao

**Affiliations:** ^1^College of Chemistry and Environmental Engineering, Shenzhen University, Shenzhen, China; ^2^Department of Gastroenterology and Hepatology, Shenzhen University General Hospital, Shenzhen, China; ^3^College of Resource and Environment, Institute of Environmental Microbiology, Fujian Agriculture and Forestry University, Fuzhou, China

**Keywords:** bromate reduction, carbon source, isolation, genome, transcription

## Abstract

Bromate, a possible human carcinogen, can be reduced to innocuous bromide by microorganisms. To characterize bromate reducers, microbes were enriched anaerobically from activated sludge by using bromate as the sole electron acceptor and different carbon sources as the electron donor. Bacteria that showed significant bromate-reducing activity but not coupled to cell growth were isolated. Two whole genomes of the isolates, namely, *Raoultella electrica* Lac1 and *Klebsiella variicola* Glu3, were reconstructed by Illumina and Nanopore sequencing. Transcriptomic analysis suggested that neither the respiratory nitrate reductase, the selenate reductase, nor the dimethylsulfoxide reductase was involved in the bromate reduction process, and strain *K. variicola* Glu3 reduced bromate *via* a yet undiscovered enzymatic mechanism. The results provide novel phylogenetic insights into bromate-reducing microorganisms and clues in putative genes encoding enzymes related to bromate reduction.

## Introduction

Halogen oxygenates, including chlorate, perchlorate, and bromate, have gained great attention mainly due to their toxicity, even at low concentrations to human health. Bromate (BrO_3_^−^) has been classified as a possible carcinogen (group 2B) by the International Agency for Research on Cancer (IARC). Once formed in aquatic environments, bromate is difficult to remove due to its high solubility and low chemical reactivity, posing a risk to aquatic organisms and public health. In drinking water that originally contains bromide, bromate is mainly formed by ozonation (Glaze et al., 1993), while in natural water, such as rivers and groundwater, bromate has been shown to accumulate through uncontrolled industrial emissions (Butler et al., 2005). Bromate concentrations in groundwater and wastewater sampled from some regions of Manila in the Philippines reached as high as 246 and 342 μg/l, respectively (Genuino and Espino, 2012). Drinking water collected from four regions of Chile showed an average concentration of 18.5 μg/l bromate, which was higher than the limit of 10 μg/l for drinking water according to the standards of the United States Environmental Protection Agency and the WHO (Raul et al., 2019). Thus, it is essential to develop efficient bromate removal technologies to prevent potential risks. Similar to the microbial reduction of perchlorate to chloride (Kim and Logan, 2001; Brown et al., 2002), microbial reduction of bromate to innocuous bromide could be a promising approach to remove bromate from the contaminated water.

To date, although the biological reduction of bromate has been realized in many types of bioreactors, and several attempts have been made to identify bromate-reducing consortia, the phylogenetic characterization of bromate reducers remains inconclusive (Assunção et al., 2011; Davidson et al., 2011; Liu et al., 2012; Zhong et al., 2018). Moreover, although some bromate-reducing bacterial strains that belong to *Actinobacteria, Bacteroidetes, Firmicutes, Alphaproteobacteria, Betaproteobacteria,* and *Gammaproteobacteria* have been isolated and identified in previous studies (Hijnen et al., 1995; Kirisits et al., 2002; Davidson et al., 2011; Tamai et al., 2016), the specific bromate-reducing pathway has remained unclear. Thus, our work aims to isolate a number of differing bromate-reducing bacteria and to uncover genomic insights into bromate reducers. The results may deepen our understanding toward the metabolic pathways of microbial bromate reduction.

## Materials and Methods

### Sampling and Reactor Operation

To enrich the bromate-reducing consortia, seed sludge was inoculated with the aerobic activated sludge of the Futian STP located in Shenzhen, China. Potassium bromate was used as the sole electron acceptor, and 0.50 g/l sodium acetate, glucose, and sodium lactate were separately added as electron donors in the reactors, namely, RA, RG, and RL, respectively (Supplementary Table S1). One liter of the medium contained 1.60 g K_2_HPO_4_, 0.86 g KH_2_PO_4_, 1.00 g (NH_4_)_2_HPO_4_, and 1 ml trace element solution (TES; Supplementary Table S2). The final pH of the medium was kept at 7.2 and regularly monitored using a compact LAQUAtwin-pH-11 pH meter (HORIBA, Japan). The pH of the medium was continuously maintained by a phosphate buffer throughout the experiment, and thus, there were no more actions needed to adjust the pH. In addition, amphotericin B was added at a final concentration of 2.5 μg/ml to prevent fungal growth during enrichment. In the early stage of enrichment, the dosage of bromate in each reactor increased from 2.35 μmol/l to 39.09 μmol/l and was maintained at 39.09 μmol/l after the 5th cycle of enrichment by inoculation of enriched sludge into fresh medium. A total number of 12 cycles were performed under the enrichment experiment.

Serum bottles with a working volume of 500 ml were used as batch reactors. At each cycle, the sludge from the last cycle and fresh sterilized medium solution were filled into serum bottles with a volume ratio of 1:19, and then high-purity (99.999%) nitrogen gas was used to purge the bottles for at least 20 min to remove the residual dissolved oxygen, followed by sealing with rubber stoppers and parafilm to keep the reactors oxygen-free. All serum bottles were operated at 25°C (to simulate conditions of room temperature) and 150 rpm on an orbital shaker. The water samples were extracted for pre- and post-cycle measurement by using 5 ml syringes with needles, followed by filtration with 0.45 μm nylon membrane filters.

### Chemical Analysis

An isolation step for pure cultures has been performed before chemical analysis, please refer to Supplementary Information: Supplementary Methods and Materials 1.1 for detail. One milliliter aliquots from each bioreactor or pure culture were collected at the designated time points and centrifuged at 14000 × g for 5 min. The supernatant from each aliquot was used for bromate and bromide quantification, while the pellet from the sample of pure cultures only was used for total protein quantification, which indicated the biomass by using the Bradford method (Bradford, 1976). Bovine serum albumin (BSA, Sigma-Aldrich, United States) was used as the standard for total protein quantification, and the Bradford method was applied for pure cultures only because complex media, such as activated sludge samples, had been shown to contain a plethora of signal-quenching molecules (e.g., humic acids) that will interfere with quantification (Le et al., 2016).

Concentrations of bromate and bromide were determined by using ion chromatography (IC, 883 Basic IC plus, Metrohm, Switzerland) with a Metrosep A Supp 7–250/4.0 column. The water sample was filtered through a 0.45 μm membrane and diluted to appropriate concentrations before IC detection. The mobile phase used for IC detection was 4 mmol/l Na_2_CO_3_ with 4% (v/v) acetonitrile, and the flow rate was set as 0.8 ml/min. The column was incubated at 45°C during IC detection. The bromate reduction rate and Br mass balance were calculated based on the detected concentrations of bromate and bromide ions by IC, as described previously (Wang et al., 2019).

### Genome Reconstruction of Bacterial Isolates and Functional Analysis

Bacterial strains were isolated from the enriched culture at the end of cycle 9 by serial dilution and repeated streaking on agar plates (see Supplementary Information: Supplementary Methods and Materials 1.1 for detail). To reconstruct the whole genome of the isolates, genomic DNA of the pure cultures was extracted using the FastDNA^®^ SPIN Kit for Soil and then qualified and quantified by agarose electrophoresis and a Qubit-3 fluorometer (Invitrogen, United States), respectively. After the quality check, a total amount of 1.5 μg genomic DNA from each bacterial strain was used for library construction and nanopore MinION sequencing. The sequencing kit was SQK-LSK108 (Nanopore, Oxford), and the sequencing flow cell was FLO-MINSP6 (Nanopore, Oxford). The FASTQ format data generated after base calling were treated by Guppy (Version 3.1.5) to remove adapter and barcode sequences. In addition, Illumina sequencing was performed by Novogene Co., Ltd. (Beijing) on a NovaSeq 6,000 platform using the PE150 strategy.

Clean reads obtained from the MinION sequencing were first assembled by Flye (2.4.1) to obtain a nearly full-length genome sequence that was used as the reference in the next step (Kolmogorov et al., 2019). Then, clean reads from the Illumina sequencing were assembled by SPAdes (v3.13.1) using the hybrid option (Antipov et al., 2016), which included the assembly results from MinION sequencing to generate the final contigs. The contigs were uploaded to a web server^1^ to calculate the average nucleotide identity (ANI) and confirm their closest phylogenetic strains at the genomic level. For strain Lac1, the genomes of type strains *Raoultella ornithinolytica* NBRC 105727, *Raoultella planticola* ATCC 33531, *Raoultella terrigena* NCTC 13038, and *Raoultella electrica* DSM 102253 were used as references, while for strain Glu3, the genome of type strain *Klebsiella variicola* DSM 15968 was chosen as the reference. All those reference genomes are available on the jspecies web server. The contigs obtained were further annotated through the RAST server version 2.0 and the National Center for Biotechnology Information (NCBI) Prokaryotic Genome Annotation Pipeline (PGAP; Overbeek et al., 2014), which combines the gene caller GeneMarkS^+^ with the similarity-based gene detection approach (Ashburner et al., 2000). In addition, protein functional classification was performed by the WebMGA server with an *e*-value cutoff of 1-e^10^ (Wu et al., 2011), and Kyoto Encyclopedia of Genes and Genomes (KEGG) pathway mapping was performed by the KEGG automatic annotation server (KAAS; Moriya et al., 2007).^2^

### Transcriptomic Analysis

#### Sampling, RNA Extraction, and Sequencing

Strain *Klebsiella variicola* Glu3 was aerobically grown in LB medium at 37°C for 24 h since growth of the strain under this condition was very fast, and the activated culture was used as the inoculum for subsequent subcultures. The residual LB medium of the activated inoculum was discarded after centrifugation at 6000× g for 5 min and followed by twice washing with the chemically defined basal medium as described in 2.1. The remaining pellets from the 2 ml LB culture were separately inoculated into serum bottles that contains 100 ml basal medium, which additionally contained 1 g/l glucose, 0.1 g/l yeast extract, and 1 ml/l TES to increase the final biomass. Initial OD_600_ of the inoculums was 0.05. A final concentration of 0.1 mM or 0.5 mM bromate was added, with a set of control group without the addition of bromate. All the reactors with different dosage of bromate were biologically prepared in triplicate and anaerobically run on 37°C by purging with high-purity nitrogen gas and sealing with rubber stoppers and parafilm, which was intended to avoid the inhibition of bromate reduction by oxygen. In addition, blank medium without inoculation, medium inoculated with the heat-killed strain *K. variicola* Glu3, and fresh cultures of strains *K. variicola* Glu3 and *R. electrica* Lac1 without a carbon source were set as controls. One milliliter of the culture was collected at designated times and centrifuged at 4°C and 12,000 × g for 2 min. The supernatant was filtered through a 0.22 μm nylon membrane filter for chemical analysis, and the pellets were immediately put into liquid nitrogen for 5 min and stored at −80°C. In addition, biomass was quantified by measuring the optical density at 600 nm using a multimode plate reader (Synergy HTX, BioTek, United States).

For RNA sequencing, the frozen cell pellets collected at 3 h after inoculation, which had a similar OD_600_ of 0.3, were transferred into a dry ice cooling package and sent to Novogene Company (Nanjing, China). Then, the total RNA was extracted and quantified. RNA quality was assessed using an Agilent 2,100 bioanalyzer (Agilent Technologies, Santa Clara, CA), and the samples with RNA integrity (RIN) > 8.0 were considered to be qualified. Ribosomal RNA was removed by using the RiboZero^™^ rRNA Removal Kit (Epicenter, Madison, United States). Strand-specific cDNA libraries were constructed with a 350 bp insert size and then sequenced using the PE150 strategy on the Illumina NovaSeq platform.

#### Quality Control and Data Processing

Quality control of the raw reads was performed by trimming adapters and removing low-quality reads (Phred score Q20 ≥ 95%) by using TrimGalore (v0.6.6).^3^ The genome file of *K. variicola* strain Glu3 (GCA_009825415.1) was downloaded from the NCBI genome database, which was used as the reference to build the genome index. Then, clean reads after quality control were mapped to the index file and reference genome to produce sam files using bowtie2 (Langmead and Salzberg, 2012), which were then converted to bam files by using samtools (v1.10; Li et al., 2009).^4^ Gene counts of each sample were calculated from the bam files by using HTSeq (v0.11.3; Anders et al., 2015).^5^ Usage of those different software could be easily conducted by following their documentations, which involves very few parameters.

#### Differential Expression Analysis

The obtained raw counts were used as input to calculate the differentially expressed genes (DEGs) using the R package DESeq2 (v1.12.3; Love et al., 2014). Genes with log_2_ (fold change) ≥ 1 or log_2_ (fold change) ≤ −1 having adjusted*-p* ≤ 0.01 were identified as upregulated or downregulated DEGs. The raw counts were treated by a regularized logarithm (rlog) transformation using DESeq2.

### Deposition of Sequences

The final assembled contigs of strains *Raoultella electrica* Lac1 and *K. variicola* Glu3 were deposited in the NCBI database under the accession numbers VJZJ00000000 and VJZH00000000, respectively. The raw RNA-seq data were deposited in the NCBI SRA database under BioProject ID PRJNA741713.

## Results and Discussion

### Bromate was Effectively Removed in an Anaerobic Bioreactor

**Figure 1:**
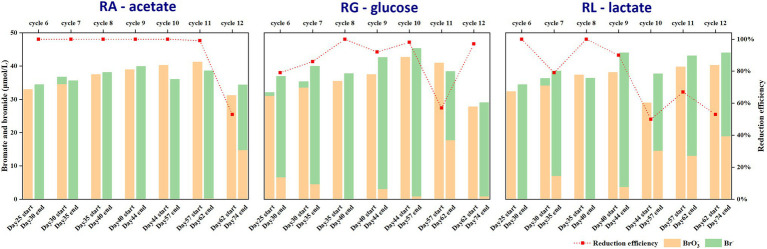
Bromate reduction rates from cycle 6 to cycle 12 in three anaerobic microbial reactors by using acetate (RA), glucose (RG), and lactate (RL) as carbon sources. The percentage of bromate reduction in the first 5 cycles was 100%.

To compare the performance of bromate reduction between the anaerobic bioreactors, bromate reduction efficiency was calculated based on the concentrations of bromate and bromide ions in the water samples before and after each enrichment cycle in the bioreactors. In the first 5 cycles (from day 0 to day 25), the initially dosed bromate (2.35–7.82 μmol/l) was completely reduced to bromide (data not shown). Then, bromate was significantly reduced to bromide in most of the following enrichment cycles. The relatively high concentration of bromate dosed in the subsequent cycles would help to enrich the potential bromate reducers. The percentage of bromate reduction was 53%—100% in RA but 57%—100 and 50%—100% in RG and RL, respectively (Figure 1). The turbidity of the sludge in the reactors gradually decreased, indicating a decreasing biomass concentration (Supplementary Figure S1), which might have resulted in the observed variation in bromate-reducing efficiency. The decreasing biomass also indicates that bromate was likely not being used as an electron acceptor for growth. Moreover, the mass balance of Br in most cycles ranged from 0.9–1.1, which accounted for 90 to 110% of the initial Br, implying that bromate was stoichiometrically reduced to bromide. Together, the results indicated the presence of bacteria that induce bromate reduction in the bioreactors. Considering the stable bromate reduction performance in different bioreactors, it appears that bromate reduction was conducted by reducing substances or by non-bromate-specific terminal reductases. In addition, our observations may also suggest the ubiquitous presence of bromate reducers able to use different electron donors.

### Isolation of Bromate-Reducing Bacteria

**Figure 2:**
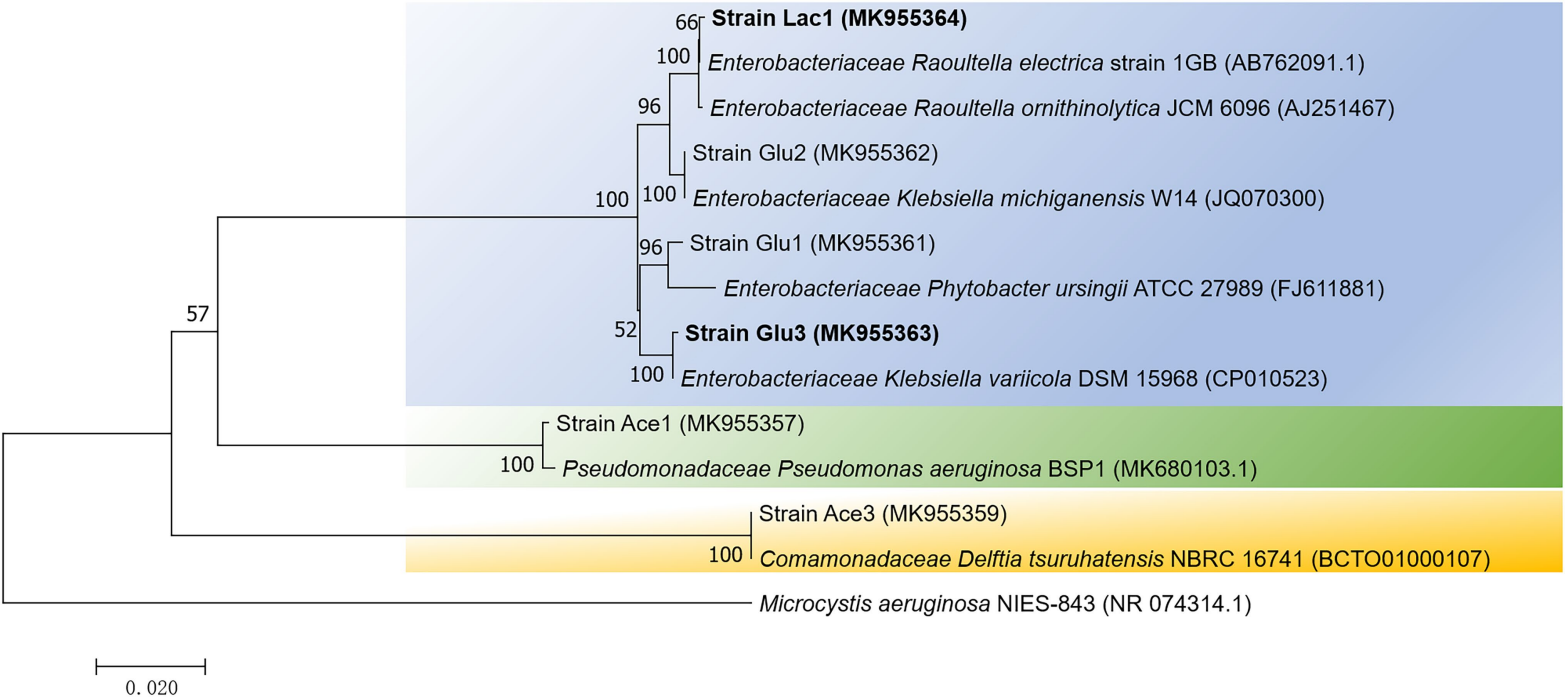
A neighbor-joining phylogenetic tree showing the relationship of the 6 isolates from enriched cultures. The tree was constructed based on 16S rRNA gene fragments with alignment lengths longer than 1,300 bp. The scale bar represents 2% sequence dissimilarity, and bootstrap values >50% are indicated at each node. *Microcystis aeruginosa,* a member of *Cyanobacteria,* was recruited as the outgroup sequence.

To further investigate the microbial strains performing bromate reduction, pure cultures were isolated from the anaerobic bioreactors after 12 cycles of enrichment (day 74). A total of 15 strains were obtained by 10-fold serial dilution and streaking. Finally, six strains were selected for further analysis according to their distinct fragment length polymorphism (RFLP) profiles (see Supplementary Information: Supplementary Materials and Methods 1.1 for details). Among them, strains Ace1 and Ace3 were isolated from RA, strains Glu1, Glu2, and Glu3 were isolated from RG, and strain Lac1 was isolated from RL. As shown in Figure 2, the 6 bromate-reducing candidate strains were classified into 5 genera: *Raoultella*, *Phytobacter*, *Klebsiella*, *Pseudomonas*, and *Delftia*. Notably, four of the six strains belonged to the family *Enterobacteriaceae*, and strains Ace 1 and Glu2 were phylogenetically closest to the pathogens *Pseudomonas aeruginosa* and *Klebsiella oxytoca*, respectively, which may limit their potential application (Lyczak et al., 2000; Hogenauer et al., 2006).

Considering that the seed sludge used for anaerobic enrichment was taken from aerobic activated sludge, the enriched bacteria were likely facultative anaerobes. Bacterial isolates belonging to the phyla *Proteobacteria*, *Firmicutes*, *Actinobacteria,* and *Bacteroidetes* have been reported to be involved in bromate reduction (Hijnen et al., 1995; Kirisits et al., 2002; Assunção et al., 2011; Davidson et al., 2011; Liu et al., 2012; Demirel et al., 2014; Zhong et al., 2018), however, to the best of our knowledge, no bromate-reducing bacteria belonging to *Enterobacteriaceae* have been identified by other groups, although other genera that belonging to *Proteobacteria*, such as *Citrobacter* (Assunção et al., 2011), *Sphingomonas* (Liu et al., 2012), and *Denitratisoma* (Demirel et al., 2014), have been reported to be involved in bromate reduction. Thus, the cultural isolates here have expanded our understanding of the potential diversity of bromate-reducing bacteria.

### Bromate-Reducing Efficiency of the Bacterial Isolates

After comparing the bromate-reducing performance between the 6 candidates, strains Lac1, Glu2, and Glu3 displayed the highest percentages of bromate removal, ranging from 74–91% (Supplementary Table S3). Considering strain Lac2 to be a potential pathogen would require special protection when being manipulated, therefore only strains Lac1 and Glu3 were selected for further evaluation.

**Figure 3:**
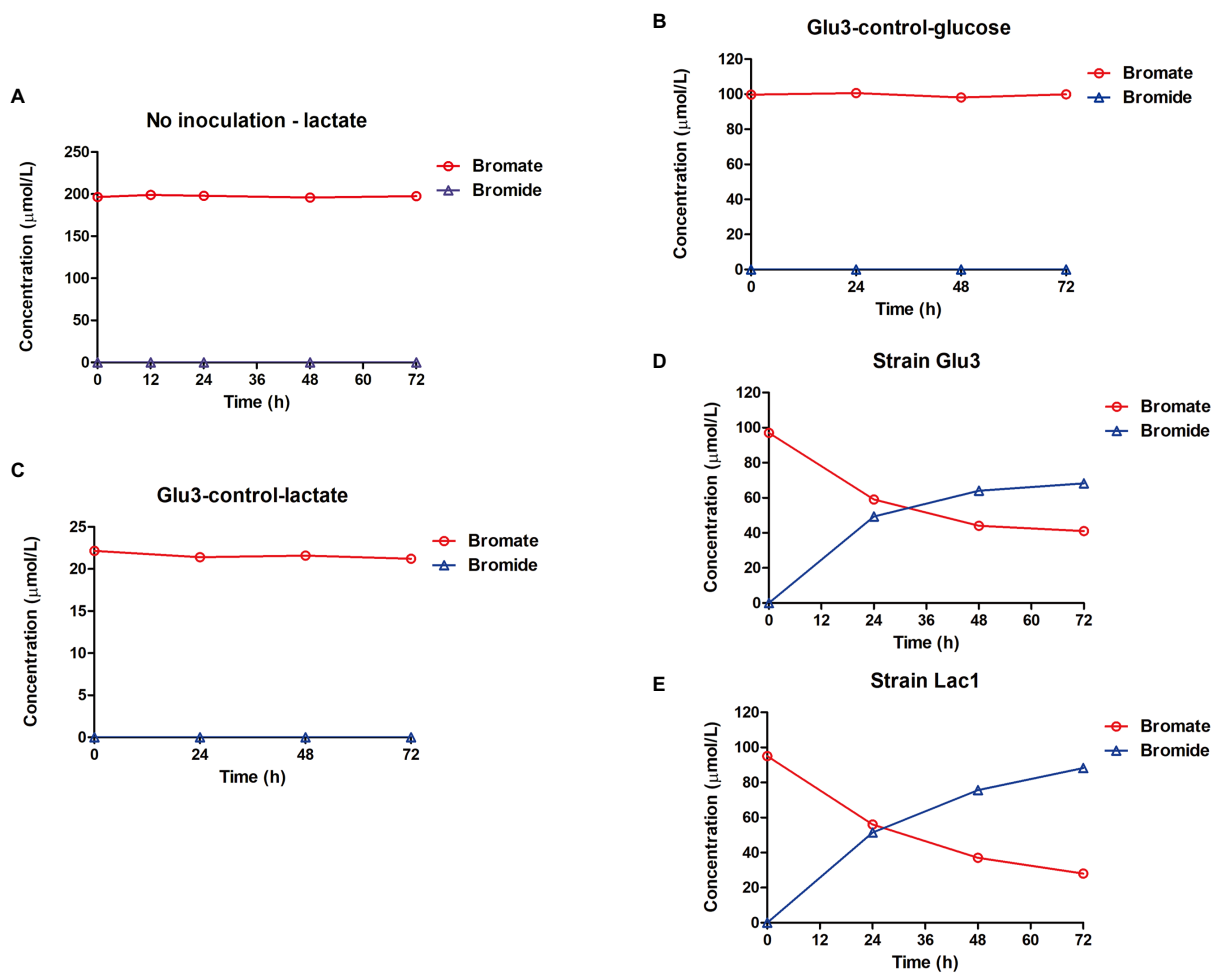
Control experiments showing bromate reduction were due to biological activity. **(A)** Mineral medium with 1 g/l lactate, 200 μmol/l bromate, and without inoculation. **(B)** Heat-killed strain *K. variicola* Glu3 incubated with 0.5 g/l glucose and 100 μmol/l bromate. **(C)** Heat-killed strain *K. variicola* Glu3 incubated with 0.5 g/l lactate and 20 μmol/l bromate. **(D)** Fresh culture of strain *K. variicola* Glu3 incubated without a carbon source and with 100 μmol/l bromate. **(E)** Fresh culture of strain *R. electrica* Lac1 incubated without a carbon source and with 100 μmol/l bromate. Error bars represent the estimated standard deviations for duplicate samples and were not visible if less than the size of the symbol.

**Figure 4:**
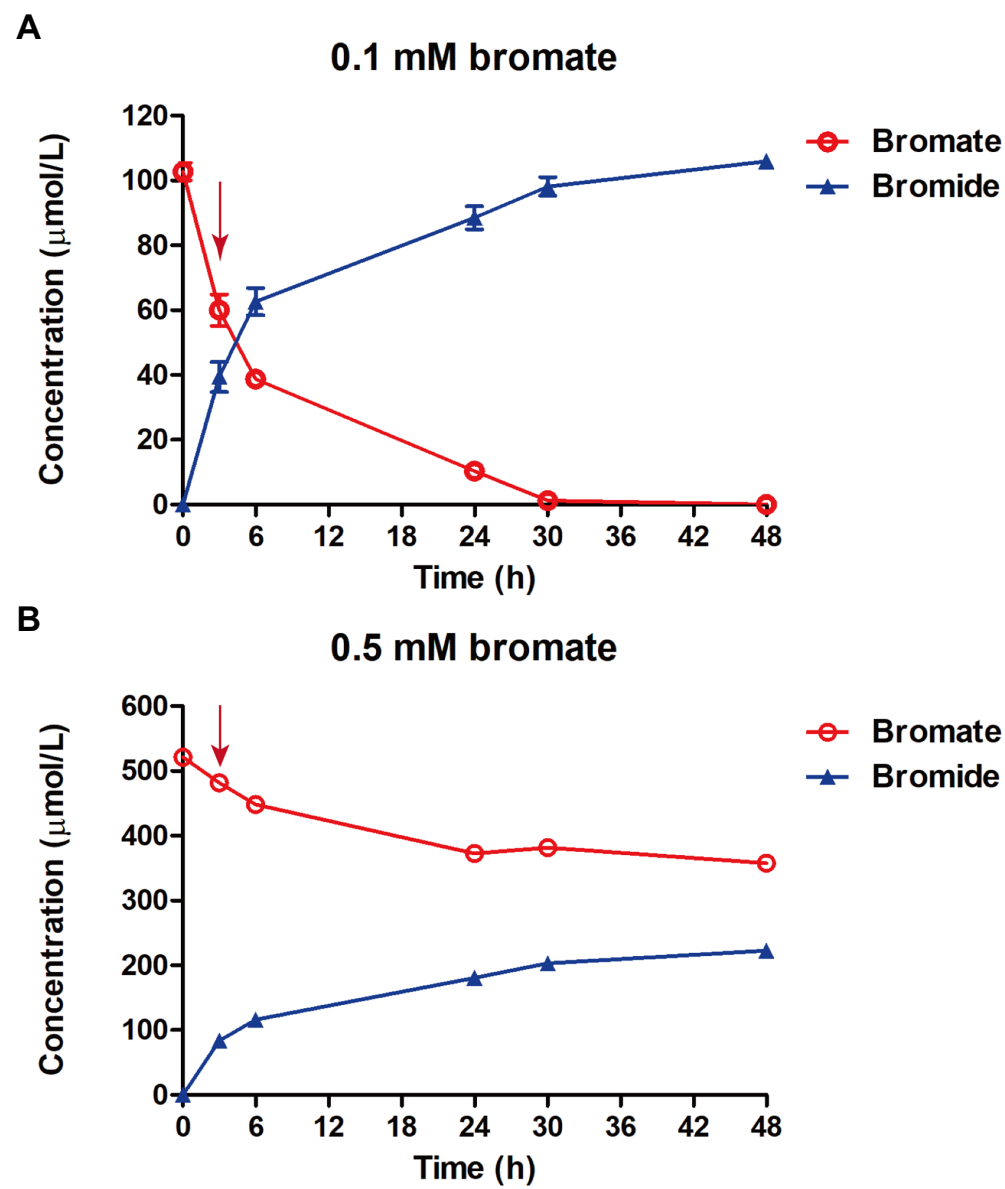
Determination of bromate reduction in strain *K. variicola* Glu3 with 0.1 mm **(A)** or 0.5 mm **(B)** of bromate dosage. Error bars represent the estimated standard deviations for triplicate samples and were not shown if less than the size of the symbol. Red arrow indicates the sampling time for RNA sequencing at 3 h after bromate dosage.

First, a set of control experimental results showed that both the blank medium dosed with bromate and medium inoculated with the heat-killed strain displayed no bromate reduction, while the medium inoculated with the cells only but without any carbon source was able to reduce considerable amounts of bromate to bromide (Figure 3). This clearly demonstrated that strains Lac1 and Glu3 were able to biologically reduce bromate. In the presence of a carbon source, such as glucose, bromate was reduced to bromide more effectively by strain Glu3 when given the same amount of initial bromate dosage (Figure 4A). In the presence of 0.5 g/l organic carbon source (the same as in the enrichment process) and 40 μmol/l or 100 μmol/l potassium bromate, both strains Lac1 and Glu3 showed no significant cell growth during the process, as indicated by the total protein quantification, which is more sensitive than the optical density method (see Supplementary Information: Supplementary Methods and Materials 1.2 for details of the experiment; the results are shown in Supplementary Figure S2). In addition, both strains displayed a higher bromate-reducing efficiency with glucose assimilation than with lactate assimilation at bromate concentrations of both 40 μmol/l and 100 μmol/l. In comparison, strain Glu3 showed an almost 2-fold higher bromate-reducing efficiency than strain Lac1 under both bromate concentrations when glucose was used as the carbon source; inversely, the bromate reduction efficiencies of strain Glu3 were lower than those of strain Lac1 when lactate was the carbon source. Under 40 μmol/l bromate exposure, when glucose was used as the carbon source, the percentages of bromate reduction for strains Lac1 and Glu3 at 5 days were 44.92 and 84.73%, respectively; however, when lactate was used as the carbon source, the corresponding percentages of bromate reduction for both strains decreased to 27.68 and 14.09%. When 100 μmol/l bromate and glucose as the carbon source, the percentages of bromate reduction for strains Lac1 and Glu3 at 5 days were 25.81 and 37.19%, respectively; however, when lactate was used as the carbon source, the corresponding percentages of bromate reduction for both strains decreased to 18.61 and 6.88% (Supplementary Figure S2). Considering the better performance of strain Glu3 in anaerobic bromate reduction, this strain was selected for further transcriptomic analysis.

### Genome Assembly and Annotation

To reconstruct the genomes of strains Lac1 and Glu3, Illumina sequencing technology combined with nanopore sequencing was applied. Illumina NovaSeq sequencing has the advantage of high accuracy for single bases, while Oxford Nanopore MinION sequencing can generate considerably longer read lengths (Lu et al., 2016). High-quality draft genomes can be reconstructed by taking advantage of these two sequencing technologies (Ruan et al., 2020). For strain Lac1, a total of 2.1 Gb and 251 Mb of clean reads were generated from Illumina sequencing and MinION sequencing, respectively. For strain Glu3, the clean reads generated from Illumina and MinION sequencing were 2.35 Gb and 1.1 Gb.

After removing contigs smaller than 200 bp, the final assembly for strain Lac1 contained 18 contigs with an N50 (with the shortest sequence length at 50% of the total genome length) value of 3,185,406 bp, while for strain Glu3, the final assembly was composed of 16 contigs with an N50 value of 4,318,936 bp. The assembly results showed that strain Lac1 contains 3 plasmids, and strain Glu3 contains 2 plasmids. The reconstructed genome sizes for strains Lac1 and Glu3 were 5.47 Mbp and 5.65 Mbp with average coverages of 327× and 389×, respectively. Based on the ANI analysis, strain Glu3 showed the highest ANI value of 98.11% with *Klebsiella variicola* DSM 19568^T^, while strain Lac1 showed an ANI value of 99.13% with the closest type strain of *Raoultella electrica* NBRC 109676^T^. Supplementary Table S4 presents the difference in the gene numbers (in percentage) in each COG category between strains Lac1 and Glu3. Again, although strain Lac1 and Glu3 appeared not to be novel species based on ANI values, it is noteworthy that no bromate-reducing activity had previously been documented for both *Klebsiella variicola* and *Raoultella electrica*.

### Transcriptomic Analysis

Strain *K. variicola* Glu3 was able to significantly reduce 0.1 mm or 0.5 mm bromate into stoichiometric amount of bromide under anaerobic condition (Figure 4). To identify putative bromate-reducing genes, transcriptomic analysis was performed using glucose as the electron donor. A total of 9 bacterial cell samples were collected after 3 h of bromate dosage. For each sample, 3,611,496–8,015,146 clean reads were obtained, and 97.93–98.44% of these reads were aligned to the genome of strain *K. variicola* Glu3 (Supplementary Table S5). Raw counts were obtained from the aligned reads by the tools described in 2.5.2. To identify putative genes responsible for reducing bromate to bromide, differential gene expression assays were performed. Total DEGs were calculated based on the normalized raw counts from last step.

The respiratory nitrate reductase purified from *Escherichia coli* K12 has been shown to have *in vitro* bromate-reducing activity (Morpeth and Boxer, 1985). In addition, a membrane-bound heterotrimeric selenate reductase purified from *Enterobacter cloacae* SLD1a-1, which contains molybdenum, heme, and non-heme iron as prosthetic constituents, also showed low reduction activity toward bromate (Ridley et al., 2006). Both the nitrate and selenate reductases belong to the dimethyl sulfoxide reductase (DMSOR) family, and it has been proposed that the DMSOR family includes enzymes with great versatility in utilizing different non-specific substrates that might include bromate (Miralles-Robledillo et al., 2019). Homologues of both the respiratory nitrate reductase encoding genes (FNX95_06385, FNX95_06390, FNX95_06395, and FNX95_06400) and the selenate reductase encoding gene (FNX95_09885) were present on the genome of strain *K. variicola* Glu3. In addition, the dimethylsulfoxide reductase encoding genes (FNX95_13385 and FNX95_13390), which is another member of the DMSOR family, were also present. However, transcription of those genes mentioned above was downregulated when 0.1 mm or 0.5 mm bromate was dosed (Table 1), indicating that those DMSOR family proteins were not involved in the bromate reduction process. In addition, transcription of the molybdopterin synthase encoding genes (FNX95_13975, FNX95_13980, FNX95_13985 and FNX95_13990) and the nitrite reductase encoding genes (FNX95_22540 and FNX95_22545) were also downregulated. Intriguingly, the transcription of *bdcA* (FNX95_10925), which encodes a NADPH-dependent short-chain dehydrogenase/reductase (SDR) oxidoreductase that may function in bacterial quorum sensing and biofilm dispersal (Lord et al., 2014), was upregulated under both 0.1 mM and 0.5 mM of bromate dosage. In addition, transcription of several genes encoding iron uptake related proteins, including the catecholate siderophore receptor CirA (FNX95_03485), two TonB-dependent siderophore receptors (FNX95_05875 and FNX95_12490), the salmochelin siderophore protein IroE (FNX95_09270), and a siderophore-interacting protein (FNX95_23825) were upregulated under both bromate dosages (Table 1). Transcription of a bacterioferritin-associated ferredoxin encoding gene (FNX95_22690) was also upregulated. However, it is not clear how the presence of bromate is related to iron uptake and delivery. A possible link is that bacteria recruit iron ions to synthesize reductases that contains iron–sulfur clusters, which thus could be used to defend and protect against the stress caused by bromate (Li et al., 2019). The highly upregulated glutaredoxin-like protein encoding gene *nrdH* (FNX95_01355, Table 1) indicated that bromate may cause oxidative stress even under anaerobic condition, since *nrdH* has been reported to enhance resistance to multiple oxidative stresses by acting as a peroxidase cofactor (Si et al., 2014). Other genes related to synthesis of cytochromes, glutathione, and sulfhydryl-associated substances were not differentially expressed under both 0.1 mM and 0.5 mM bromate exposure. Unfortunately, transcriptomic results did not indicate a specific terminal reductase that might have been responsible for bromate reduction in strain *K. variicola* Glu3. It has been reported that genes encoding functions related to type II outer membrane protein secretion and metal reduction specifically the outer membrane MtrAB module of the extracellular electron conduit MtrCAB are required for iodate reduction by *Shewanella oneidensis* (Toporek et al., 2019); however, no similar genes were differentially expressed in strain *K. variicola* Glu3.

**Table 1:** Differentially expressed genes (DEGs) with a dosage of 0.1 mm and 0.5 mm bromate in strain *K. variicola* Glu3.

Gene ID	0.1 mm vs. 0 mm log_2_FC	0.5 mm vs. 0 mm log_2_FC	Symbol	Functions
FNX95_01355	6.99	5.00	*nrdH*	glutaredoxin-like protein
FNX95_03485	4.99	2.74	*cirA*	catecholate siderophore receptor
FNX95_05875	5.38	4.66	NA	TonB-dependent siderophore receptor
FNX95_09270	2.15	2.30	*iroE*	Salmochelin siderophore protein
FNX95_10925	1.14	1.64	*bdcA*	SDR family oxidoreductase
FNX95_12490	3.19	2.61	NA	TonB-dependent siderophore receptor
FNX95_22690	2.12	2.36	NA	bacterioferritin-associated ferredoxin
FNX95_23825	1.71	1.32	NA	siderophore-interacting protein
FNX95_24530	2.61	4.82	NA	thioredoxin domain-containing protein
FNX95_24535	2.75	4.92	NA	protein-disulfide reductase
FNX95_25960	1.05	1.45	*dsbA*	thiol:disulfide interchange protein
FNX95_06385	−4.85	−2.58	*narG*	nitrate reductase subunit alpha
FNX95_06390	−3.68	−2.27	*narH*	nitrate reductase subunit beta
FNX95_06395	−3.91	−1.97	*narJ*	nitrate reductase molybdenum cofactor assembly chaperone
FNX95_06400	−3.80	−1.91	*narI*	respiratory nitrate reductase subunit gamma
FNX95_07655	−3.90	−2.85	*sodB*	superoxide dismutase [Fe]
FNX95_09885	−2.20	−1.30	NA	molybdopterin-dependent oxidoreductase
FNX95_13380	−3.16	−1.82	NA	dimethylsulfoxide reductase
FNX95_13385	−3.89	−2.21	*dmsB*	dimethylsulfoxide reductase subunit B
FNX95_13390	−3.59	−1.85	*dmsA*	dimethylsulfoxide reductase subunit A
FNX95_13975	−1.73	−1.27	*moaE*	molybdopterin synthase catalytic subunit
FNX95_13980	−2.03	−1.34	*moaD*	molybdopterin synthase sulfur carrier subunit
FNX95_13985	−1.78	−1.43	*moaC*	cyclic pyranopterin monophosphate synthase
FNX95_13990	−1.94	−1.47	*moaB*	GTP 3,8-cyclase
FNX95_14335	−1.53	−1.32	*ybgE*	cyd operon protein
FNX95_14345	−1.38	−1.61	*cydB*	cytochrome d ubiquinol oxidase subunit II
FNX95_14350	−1.31	−1.50	*cydA*	cytochrome d ubiquinol oxidase subunit I
FNX95_22540	−3.67	−1.63	*nirD*	nitrite reductase small subunit
FNX95_22545	−3.35	−1.39	*nirB*	nitrite reductase large subunit

In the bromate-reducing bacterium *Rhodococcus* sp. Br-6, both biotic and abiotic reactions were shown to be involved in bromate reduction in the presence of electron mediators including ferric iron and 2,6-dichloroindophenol (DCIP; Tamai et al., 2016). Similarly, bromate could also be reduced to bromide by sulfide which is biologically produced through microbial sulfur disproportionation (Chairez et al., 2010). These observations gave a hint as to the diversity of bromate-reducing pathways by different bacteria. Transcriptomic results showed that transcription of the gene encoding sulfite reductase (FNX95_00780) was not significant. In addition, since only a trace amount of sulfate or other sulfur-containing compounds were added to the medium, there was a negligible possibility for the bacteria to produce sufficient sulfide for bromate reduction. Furthermore, since neither homologs to DCIP reductase nor ferric ion reductase encoding genes were found in the genome of strain *K. variicola* Glu3, and electron mediators, such as ferric iron and DCIP, were not added under our experimental conditions, it is unlikely that the enriched or pure cultures utilize a similar bromate-reducing pathway as shown in strain *Rhodococcus* sp. Br-6 (Tamai et al., 2016).

In summary, bromate-reducing bacteria were enriched from the activated sludge of a STP by using different kinds of carbon sources. Two bacterial isolates belonging to *Enterobacteriaceae* showed significant bromate-reducing activity, but growth of these two strains was not supported by bromate reduction. Previously reported DMSOR family reductases including the respiratory nitrate reductase and selenate reductase have shown *in vitro* bromate reduction activity in other studies, however, transcriptomic analysis suggested that they were not involved in the anaerobic bromate reduction process in strain *K. variicola* Glu3, thus indicating this strain reduces bromate *via* a yet undiscovered enzymatic mechanism. The results provide novel phylogenetic insights into bromate-reducing microorganisms and clues into putative genes correlated to bromate reduction. The bromate-reducing isolates could be good templates to uncover the metabolic mechanism of bromate reduction, and further explore bromine circulation in the biosphere.

## Data Availability Statement

The datasets presented in this study can be found in online repositories. The names of the repository/repositories and accession number(s) can be found at: https://www.ncbi.nlm.nih.gov/, PRJNA741713, VJZJ00000000, and VJZH00000000.

## Author Contributions

DW: conceptualization, methodology, investigation, and writing–original draft preparation. YW: investigation and visualization. XL: investigation. XC: methodology. WI: writing–review and editing. BY: supervision. DZ: investigation. CR: writing–review and editing. YM: conceptualization, supervision, and writing–review and editing. All authors contributed to the article and approved the submitted version.

## Funding

This study was funded by the Postdoctoral Research Foundation of China (2019M653059); The Stable Support Program of Colleges and Universities in Shenzhen (20200813153536001); and Natural Science Foundation of Shenzhen University (860–000002110245).

## Conflict of Interest

The authors declare that the research was conducted in the absence of any commercial or financial relationships that could be construed as a potential conflict of interest.

## Publisher’s Note

All claims expressed in this article are solely those of the authors and do not necessarily represent those of their affiliated organizations, or those of the publisher, the editors and the reviewers. Any product that may be evaluated in this article, or claim that may be made by its manufacturer, is not guaranteed or endorsed by the publisher.

## Footnotes

^1^ http://jspecies.ribohost.com/jspeciesws/#analyze

^2^ https://www.genome.jp/tools/kaas/

^3^ https://github.com/FelixKrueger/TrimGalore

^4^ https://github.com/samtools/samtools

^5^ https://github.com/simon-anders/htseq
