# Supplementary material for: Isolation of Anaerobic Bromate-Reducing Bacteria Using Different Carbon Sources and Transcriptomic Insights From Klebsiella variicola Glu3
Source: Front Microbiol. 2022 Mar 29;13:851844. doi: 10.3389/fmicb.2022.851844 (PMC9002608; doi:10.3389/fmicb.2022.851844)
Supplement: Supplementary file 1 [file Data_Sheet_1.docx]

**Supplementary information for**

**Isolation of anaerobic bromate-reducing bacteria using different carbon sources and transcriptomic insights from *Klebsiella variicola* Glu3**

Dan Wang^1^, Yicheng Wang^1^, Xinyue Lv^1^, Xunchao Cai^1, 3^, Waheed Iqbal^1^, Bo Yang^1^, Dan Zhou^2^, Christopher Rensing^2^, Yanping Mao^1*^

^1^College of Chemistry and Environmental Engineering, Shenzhen University, Shenzhen 518060, P. R. China

^2^Institute of Environmental Microbiology, College of Resource and Environment, Fujian Agriculture and Forestry University, Fuzhou 350002, P.R. China

^3^Department of Gastroenterology and Hepatology, Shenzhen University General Hospital, Shenzhen 518071, P.R. China

*Corresponding author: Tel: +86-755-26558094; E-mail address: maoy@szu.edu.cn (Y. Mao)

*Frontiers in Microbiology*

The supplementary information contains 5 tables, 4 figures, supplementary materials and methods and 12 pages in total.

**Table and Figure Legends**

Table S1. Running information on the anaerobic bromate reducing bioreactors.

Table S2. Composition of the trace element solution.

Table S3. Bromate reducing efficiencies at day 5 of the 6 isolated strains.

Table S4. Percentage of genes associated with the 25 general COG functional categories.

Table S5. Number of reads from RNA sequencing and alignment percentage to the reference genome of strain *K. variicola* Glu3.

Figure S1. Turbidity changes of the anaerobic bioreactors during the enrichment process.

Figure S2. Determination of bacterial growth and bromate reducing efficiency in strains *R. electrica* Lac1 and *K. variicola* Glu3.

**Supplementary tables**

Table S1. Running information of the anaerobic bromate reducing bioreactors.

| Reactor | Seed sludge type | Carbon source | Bromate concentration added in each cycle (μmol/L) | | | | | |
| --- | --- | --- | --- | --- | --- | --- | --- | --- |
|  |  |  | cycle 1 | cycle 2 | cycle 3 | cycle 4 | cycle 5 | cycle 6-10 |
| RA | AS | acetate | 2.35 | 2.35 | 3.91 | 3.91 | 7.82 | 39.09 |
| RG | AS | glucose | 2.35 | 2.35 | 3.91 | 3.91 | 7.82 | 39.09 |
| RL | AS | lactate | 2.35 | 2.35 | 3.91 | 3.91 | 7.82 | 39.09 |

Abbreviation: AS, activated sludge.

Table S2. Composition of the trace element solution.

| **Chemicals** | **Concentration (mg/L)** |
| --- | --- |
| MgSO_4_ | 24.00 |
| ZnSO_4_∙7H_2_O | 2.00 |
| FeSO_4_ | 3.80 |
| MnSO_4_∙H_2_O | 1.00 |
| Na_2_MoO_4_∙2H_2_O | 0.20 |
| Na_2_SeO_3_ | 0.10 |
| NiSO_4_∙6H_2_O | 0.10 |
| CuSO_4_∙5H_2_O | 0.29 |
| H_3_BO_3_ | 0.60 |
| D-Pantothenic acid | 3.60 |
| Na_2_EDTA∙2H_2_O | 7.00 |

Table S3. Bromate reducing efficiencies at 5 days of the 6 isolated strains. Only one of the results were shown from two independent experiments.

| Strain | Taxonomy | Similarity of 16S rRNA gene sequence | Carbon source  (0.5 g/L) | Bromate dosage (μmol/L) | Br mass balance | Bromate reducing efficiency |
| --- | --- | --- | --- | --- | --- | --- |
| Ace1 | *Pseudomonas aeruginosa* | 100% | acetate | 46.64 | 1.09 | 18% |
| Ace3 | *Delftia tsuruhatensis* | 100% | acetate | 46.64 | 1.07 | 35% |
| Glu1 | *Phytobacter ursingii* | 98.82% | glucose | 42.73 | 1.07 | 71% |
| Glu2 | *Klebsiella michiganensis* | 99.63% | glucose | 42.73 | 1.02 | 77% |
| Glu3 | *Klebsiella variicola* | 99.64% | glucose | 42.73 | 1.06 | 74% |
| Lac1 | *Raoultella electrica* | 99.52% | lactate | 45.08 | 1.05 | 91% |

Table S4. Percentage of genes associated with the 25 general COG functional categories from strain Lac1 and Glu3. The percentage of genes are calculated by the number of genes in each category divided by the total number of non-redundant genes in each strain.

| Code | Description | Strain Lac1 | Strain Glu3 |
| --- | --- | --- | --- |
| J | Translation, ribosomal structure and biogenesis | 5.32 | 5.04 |
| A | RNA processing and modification | 0.04 | 0.03 |
| K | Transcription | 9.05 | 9.54 |
| L | Replication, recombination and repair | 4.00 | 3.63 |
| B | Chromatin structure and dynamics | 0.04 | 0.02 |
| D | Cell cycle control, cell division, chromosome partitioning | 0.94 | 0.87 |
| Y | Nuclear structure | 0.00 | 0.00 |
| V | Defense mechanisms | 1.27 | 1.61 |
| T | Signal transduction mechanisms | 4.33 | 4.58 |
| M | Cell wall/membrane/envelope biogenesis | 5.11 | 5.48 |
| N | Cell motility | 0.72 | 0.87 |
| Z | Cytoskeleton | 0.00 | 0.00 |
| W | Extracellular structures | 0.00 | 0.00 |
| U | Intracellular trafficking, secretion, and vesicular transport | 1.78 | 1.90 |
| O | Posttranslational modification, protein turnover, chaperones | 3.67 | 3.56 |
| C | Energy production and conversion | 8.01 | 8.23 |
| G | Carbohydrate transport and metabolism | 11.05 | 11.03 |
| E | Amino acid transport and metabolism | 13.52 | 13.30 |
| F | Nucleotide transport and metabolism | 2.99 | 2.57 |
| H | Coenzyme transport and metabolism | 5.64 | 5.20 |
| I | Lipid transport and metabolism | 2.85 | 3.13 |
| P | Inorganic ion transport and metabolism | 8.10 | 7.40 |
| Q | Secondary metabolites biosynthesis, transport and catabolism | 2.48 | 2.77 |
| R | General function prediction only | 10.78 | 11.72 |
| S | Function unknown | 8.33 | 8.76 |

Table S5. Number of reads from RNA sequencing and alignment percentage to the reference genome of strain Glu3.

| Sample name | Sample name submitted to NCBI SRA | Number of reads | Percentage of alignment (%) |
| --- | --- | --- | --- |
| 0 mM_1 | Glu3_0mM_1 | 6,486,134 | 98.14 |
| 0 mM_2 | Glu3_0mM_2 | 3,611,496 | 97.93 |
| 0 mM_3 | Glu3_0mM_3 | 6,346,970 | 98.15 |
| 0.1 mM_1 | Glu3_0.1mM_1 | 8,015,146 | 98.26 |
| 0.1 mM_2 | Glu3_0.1mM_2 | 3,825,797 | 98.31 |
| 0.1 mM_3 | Glu3_0.1mM_3 | 6,731,361 | 98.30 |
| 0.5 mM_1 | Glu3_0.5mM_1 | 6,890,654 | 98.35 |
| 0.5 mM_2 | Glu3_0.5mM_2 | 6,411,905 | 98.42 |
| 0.5 mM_3 | Glu3_0.5mM_3 | 7,459,373 | 98.44 |

**Supplementary figures**


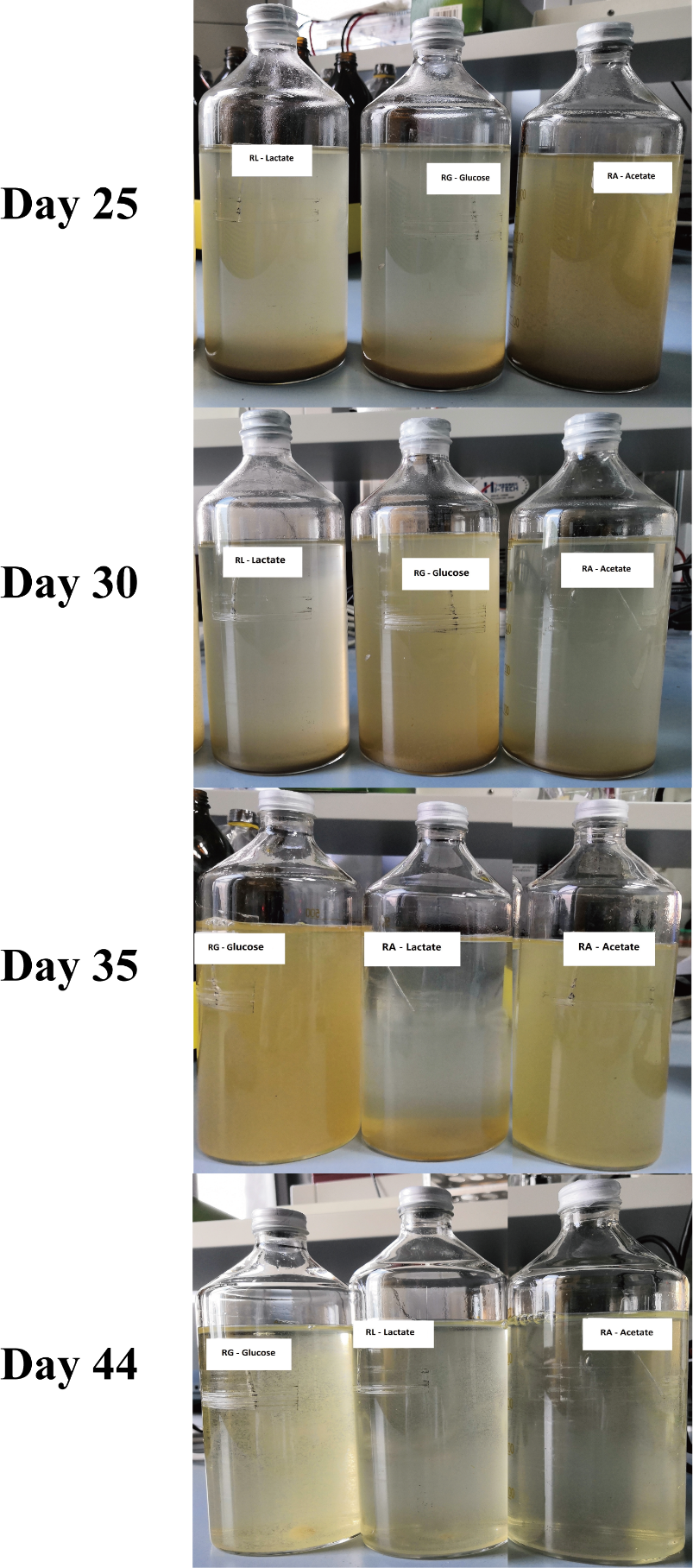


Figure S1. Turbidity changes of the anaerobic bioreactors during the enrichment process.


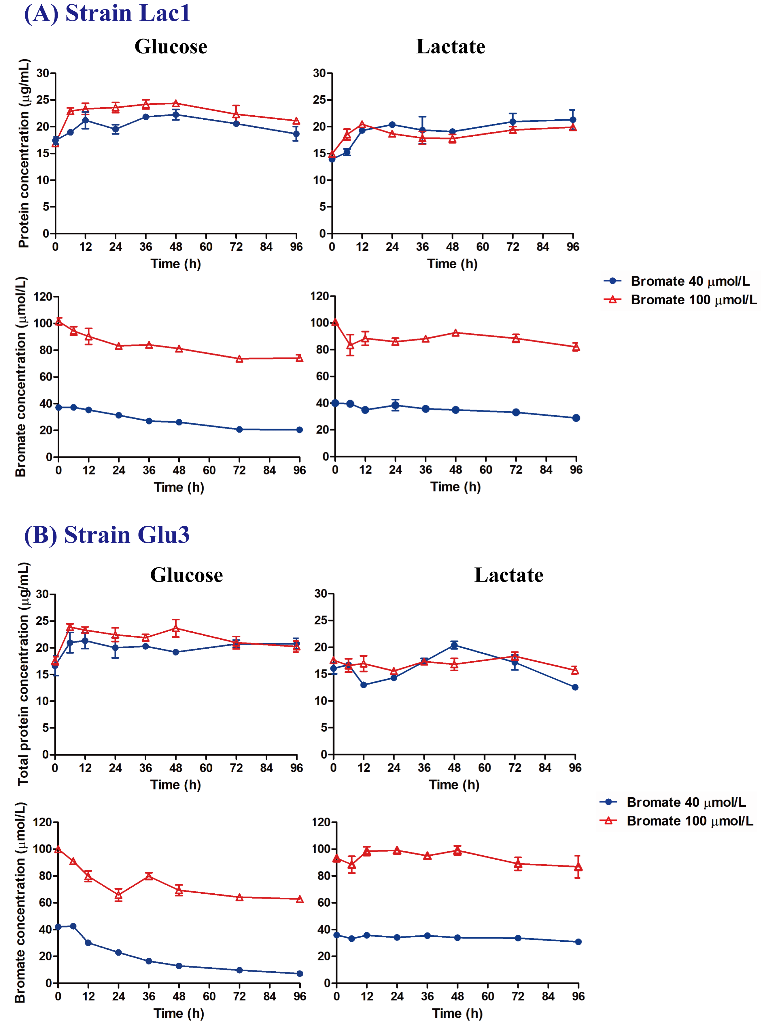


Figure S2. Determination of bacterial growth and bromate reducing efficiency in strains *R. electrica* Lac1 (A) and *K. variicola* Glu3 (B). Glucose and lactate were used as carbon sources, respectively. The initial dosage of bromate was set as 40 μmol/L and 100 μmol/L for comparison. Error bars represent the estimated standard deviations for triplicate samples and were not shown if less than the size of the symbol.

**Supplementary materials and methods**

**1.1 Bacterial strains isolation and species classification**

Bacterial strains were isolated from the enriched culture in the 3 anaerobic bioreactors using 10-fold serial dilution and spreading strategy. The agar plates used for isolation contained the same carbon source as dosed in specific bioreactors. The whole isolation process was performed in an anaerobic chamber equipped with a constant temperature incubator (Longyue LAI-3D, Shanghai, China). Once visible colonies appeared on the plate, they were separately streaked onto a new plate using a sterilized stick and then incubated at 28℃. The streaking process was repeated at least 3 times to ensure pure cultures were obtained. Since the obtained pure cultures could also aerobically grow in Luria-Bertani (LB) medium, cultures harvested from the liquid LB medium were collected and mixed with a one-quarter volume of sterilized glycerol and stored at −80°C for long term storage.

Nearly full length of 16S rRNA genes from the isolates were obtained by PCR amplification using a universal bacterial primer set of 8F/1492R. For the 16S rRNA gene amplification, a 50 μL mixture containing 25 μL 2 × PCR Master Mix, 1 μL of each primer (10 μM), 23 μL sterilized water and a picked colony was placed on a thermal cycler (T100, Bio-Rad, USA). The samples were initially denatured at 95°C for 5 min, followed by 30 cycles of denaturation at 95°C for 30 s, annealing at 50°C for 30 s, extension at 72°C for 90 s, following a final extension at 72°C for 10 min. The PCR products were digested by *Hae*III at 37℃ on a water bath for 2 h. Then, the redundant colonies were removed and the strains showed distinct fragment length polymorphism (RFLP) profiles were sent for 16S rRNA genes sequencing.

**1.2 Anaerobic growth and bromate reduction of strains *R. electrica* Lac1 and *K. variicola* Glu3**

Aerobically grown strains of *R. electrica* Lac1 and *K. variicola* Glu3 were inoculated into a medium containing 0.50 g/L carbon source, 1.60 g/L K_2_HPO_4_, 0.86g/L KH_2_PO_4_, 1.00g/L (NH_4_)_2_HPO_4_, and 1 mL/L TES. In addition, potassium bromate solution was dosed to a final concentration of 40 μmol/L or 100 μmol/L. The initial OD_600_ for both strains was 0.05. Each strain with different carbon sources were inoculated in triplicate. The serum bottles were purged with high-purity nitrogen gas and sealed prior incubating at 28℃ with shaking at the speed of 150 r/min. Samples were taken at designated time points. The serum bottles were purged with high-purity nitrogen gas and sealed again after each sampling.
